# What You See Is Not What You Get: Frontal Sinus Metastasis From the Renal Cell Carcinoma

**DOI:** 10.7759/cureus.49323

**Published:** 2023-11-24

**Authors:** Pir Abdul Ahad Aziz Qureshi, Reynir Hans Reynisson, Maríanna Garðarsdóttir, Peer Asad Aziz Qureshi

**Affiliations:** 1 Department of Radiology, Landspítali - The National University Hospital of Iceland, Reykjavík, ISL; 2 Department of Neurosurgery, Bilawal Medical College, Jamshoro, PAK

**Keywords:** renal cell cancer metastasis, mri in brain sol, metastasis, frontal sinus metastasis, renal cell carcinoma (rcc)

## Abstract

Metastatic neoplasms occurring in the nasal and paranasal sinuses are infrequent occurrences. In this study, we present one such case of a 61-year-old male patient with a known clear cell renal carcinoma presenting to us with signs and symptoms of acute sinusitis. The patient subsequently underwent CT and MRI examinations, which revealed a neoplastic mass in the right frontal sinus, which was surgically resected and was later confirmed histologically as a metastatic deposit from clear cell renal carcinoma. The patient is currently being treated with chemotherapy and radiotherapy and is doing well.

## Introduction

Renal cell carcinoma (RCC) is one of the most common malignancies in the Western world, with an annual incidence of approximately 35,000 cases in the United States [1]. It commonly affects male patients between fourth and sixth decades [1]. The patients usually present with complaints of hematuria, flank pain, and the presence of a palpable abdominal mass [1], and approximately 30% of the cases diagnosed with RCC have metastatic disease [2]. The most common sites of metastases from the RCC are to the lungs (75%), followed by soft tissues (36%), and bones and liver (20% and 18%, respectively) [1]. However, approximately 15% of the RCC metastasize to the head and neck region, predominantly to the paranasal sinuses, jaws, larynx, and parotid and thyroid glands [3].

Nevertheless, RCC is recognized as the predominant malignancy that exhibits metastatic spread to this anatomical location [1]. Clinically, these patients present with symptoms of nasal obstruction or epistaxis as a result of the highly vascular nature of the mass. We herein present the case of a 61-year-old patient diagnosed with clear cell carcinoma of the right kidney and subsequently developed right frontal sinus metastasis.

## Case presentation

A 61-year-old male with a known case of clear cell carcinoma of the right kidney presented to us in April 2022 with acute complaints of headache, fever, nasal discharge, bulging of the right eye, and a feeling of pressure behind the eyes. On a general physical examination, the patient had a fever of 100.5°F with right-sided facial swelling, erythema, and right eye proptosis. Considering the diagnosis of acute sinusitis, the patient was referred for a CT scan.

**Figure 1:**
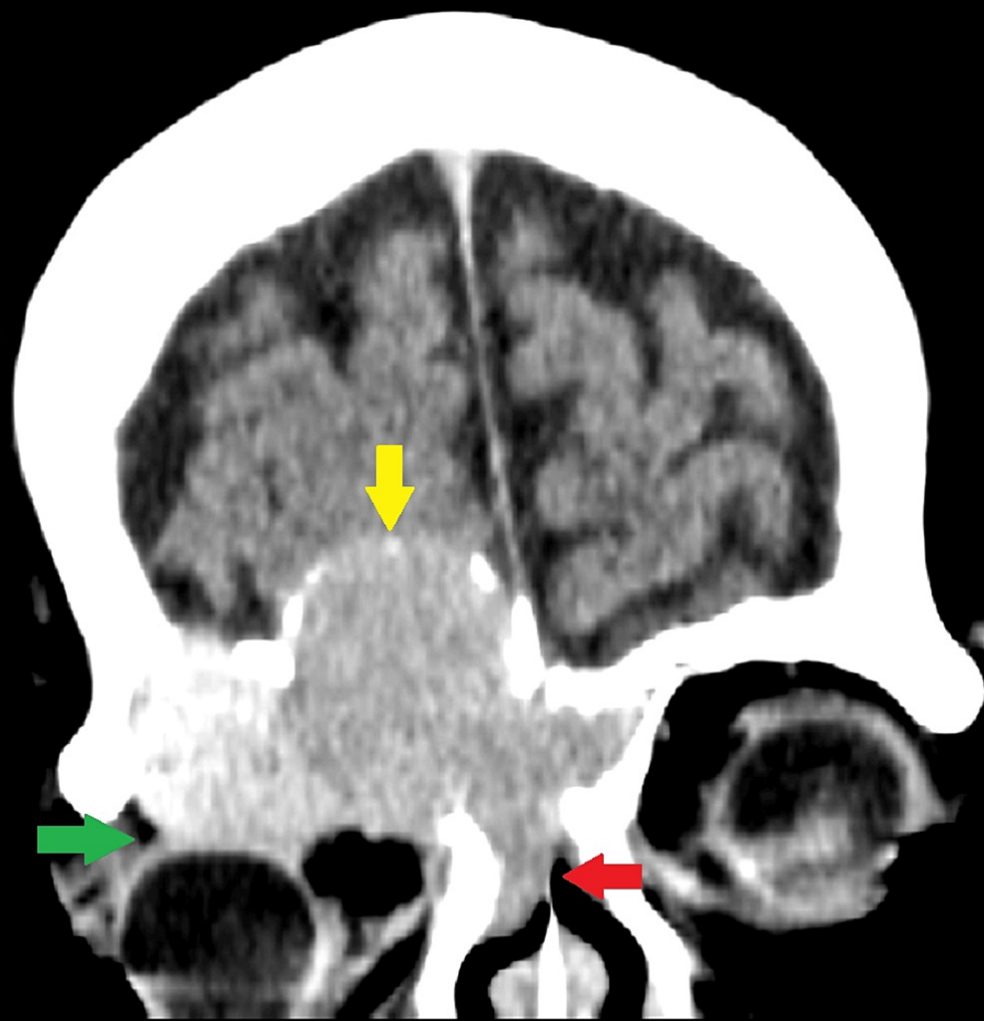
CT brain (plain) coronal view Large, heterogeneous, predominantly hyperdense mass in the right frontal sinus, destroying the walls of the right frontal sinus, resulting in compression of the right frontal lobe (yellow arrow). The mass also extends into the ethmoid sinus (red arrow) and the right orbit (green arrow), causing proptosis of the right eyeball.

Subsequently, the patient underwent a CT scan, which showed a large, heterogeneous, predominantly hyperdense mass in the right frontal sinus, destroying the posterior wall of the right frontal sinus and compression of the right frontal lobe. There was also mild opacification of the left frontal sinus and bilateral ethmoid sinuses (Figure 1).

**Figure 2:**
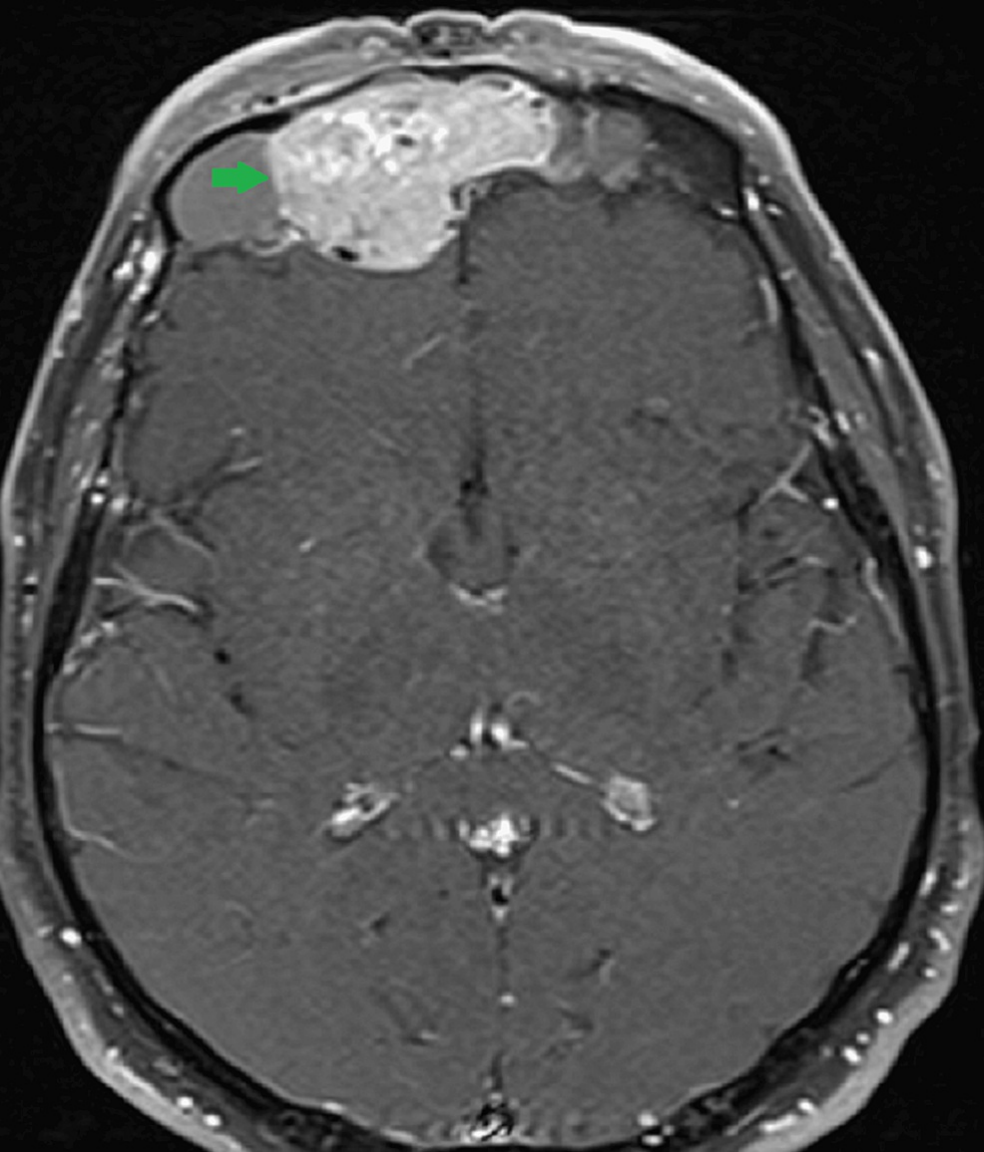
MRI brain (axial, T1 post-contrast) Large enhancing mass in the right frontal sinus (green arrow) destroying the posterior wall of the right frontal sinus with extension in the anterior cranial fossa, closely abutting and compressing the frontal lobe.

For further evaluation of the mass, the patient underwent an MRI with contrast, confirming the presence of a large mass showing T1 hypointense and T2 intermediate signals and post-contrast enhancement. The mass was seen to extend into the left frontal sinus and anterior ethmoid air cells. It was destroying the posterior wall of the right frontal sinus, with extension in the anterior cranial fossa closely abutting and compressing the frontal lobe. Few signal voids were seen within it, suggesting blood vessels. Inferiorly, the mass was also extending into the extraconal compartment of the right orbit, abutting the right eyeball, resulting in proptosis of the right eye (Figure 2).

Bilateral optic nerves were normal. No fat stranding was seen in the intraconal compartment of both orbits. Based on CT and MRI findings, a frontal sinus neoplastic mass was diagnosed.

The patient was then referred for discussion at the multidisciplinary tumor board meeting, which recommended undergoing surgical resection of the mass followed by chemotherapy and radiotherapy. Subsequently, the tumor was removed from the right frontal sinus by performing a craniotomy. Initially, two burr holes were made just above where the frontal sinus ends in the midline and two to the sides behind the junctions of the lateral and superior parts of the orbit. Then the burr was loosened, and then a large bone plate was sawn. The frontal sinus was then accessed, the tumor was removed, and the dura was freed from the inside of the skull. The resected mass was then sent for histopathological evaluation to confirm the diagnosis, which confirmed that the neoplastic mass was compatible with metastasis from a clear cell carcinoma.

**Figure 3:**
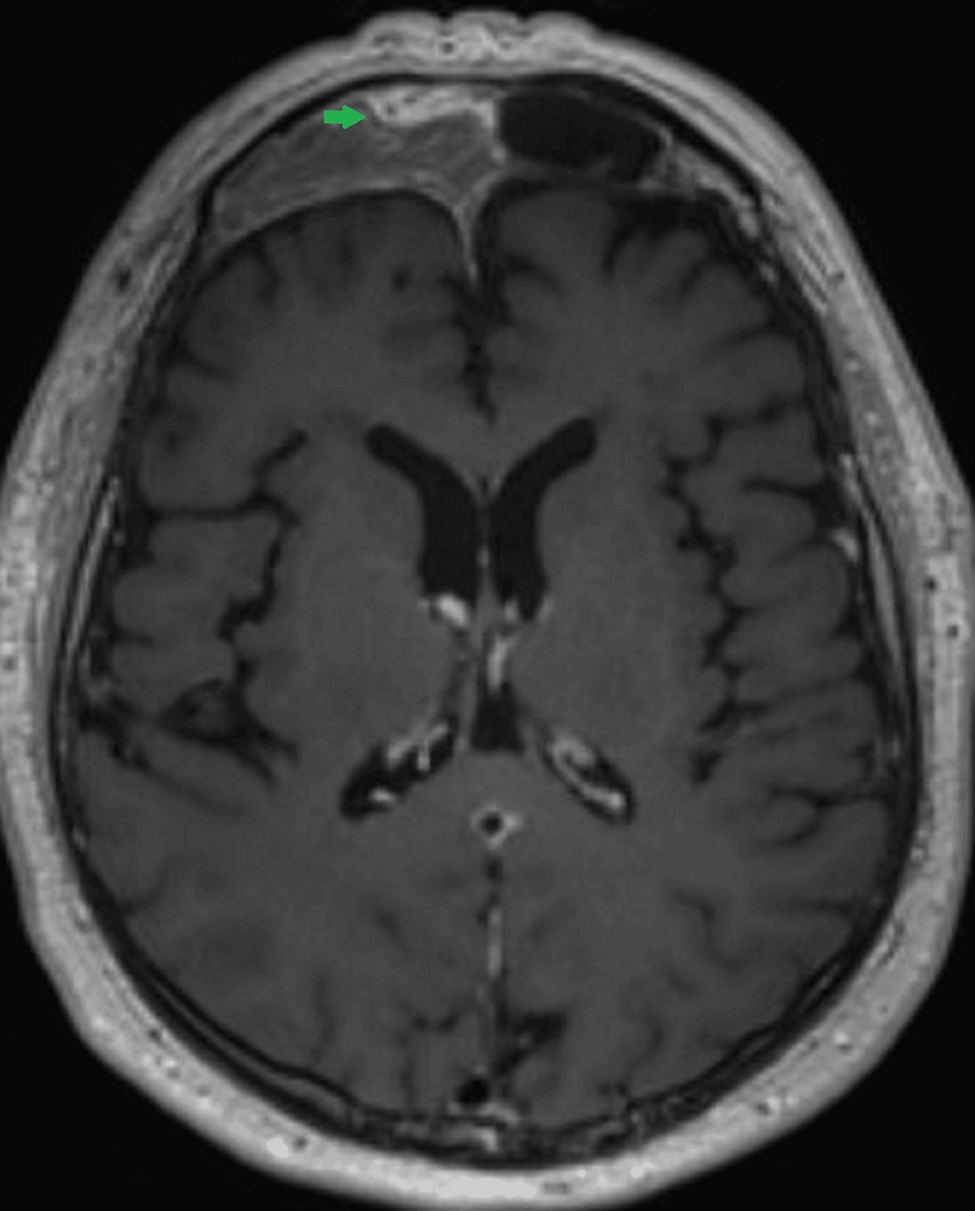
MRI brain (axial, T1 post-contrast) Small residual enhancing tumor in the right frontal sinus (green arrow).

On the three-month post-chemotherapy and radiotherapy follow-up MRI, there was a small residual tumor in the right frontal sinus (Figure 3).

The patient was continued on chemotherapy, and the latest follow-up MRI performed in September 2023 showed stable appearances of the frontal sinus.

## Discussion

The malignancies originating in the nasal cavity and paranasal sinuses are usually considered primary tumors. However, metastases to the paranasal sinuses can be rarely seen. RCC, being well-known for its tendency to metastasize to unusual sites, is among the most common tumors that metastasize to the paranasal sinuses, accounting for 49% of these cases, followed by tumors arising from the bronchus, urogenital, breast, and gastrointestinal tract [4]. Matsumoto and Yanagihara (1982) were the first to publish a report in the literature describing the renal clear cell carcinoma metastasizing to the paranasal sinuses [1]. Subsequently, RCC metastases to the head and neck region were reported by several authors. Homer and Jones reported RCC metastasis as a solitary periorbital mass [5], whereas Jung et al. reported RCC metastasis to the left orbit and ethmoid sinus [6], and Treada et al. reported RCC metastasis to the ethmoid sinuses [7].

The tumor cells originating from RCC have the ability to migrate to the sinonasal region by two distinct pathways. The first pathway involves the inferior vena cava, lungs, heart, and maxillary artery. The second pathway entails the communication between the avalvular vertebral venous plexus and the intracranial venous plexus. Metastatic tumors exhibit a predilection for the maxillary sinuses (36%), followed by the ethmoid sinuses (25%). The frontal and sphenoid sinuses and the nasal cavity are affected to a lesser extent (17% and 11%, respectively) [1].

Differential diagnosis is crucial when encountering sinonasal metastasis from RCC, as it necessitates distinguishing it from primary tumors like adenocarcinomas, angiofibromas, hemangiopericytomas, melanomas, hemangiomas, metastatic tumors originating from the breast and lungs, and, less commonly, systemic diseases such as granulomatosis with polyangiitis and midline granulomas. Hematuria, however, may serve as a potential signal of RCC, as studies have revealed that approximately 10% of individuals diagnosed with RCC and distant metastases present with significant hematuria. Nevertheless, it is worth noting that in 90% of instances, intermittent hematuria may be observed. Therefore, it is imperative that patients who come with nasosinusal tumors with hematuria undergo a comprehensive systemic assessment. To comprehensively evaluate the scope of the metastatic lesion, it is imperative to do radiological assessments utilizing a CT scan as the primary modality, followed by MRI and angiography. However, the definitive diagnosis is typically established after the surgical extraction of the metastatic tumor, followed by the histological analysis of the tissue [1].

It is essential to acknowledge that RCC metastases exhibit radiographic characteristics that resemble those of original malignant lesions in the sinonasal cavities. Certain indications of renal origin of such masses observed on CT scans are enhancement, destruction, and the absence of intra-tumoral calcification [1]. The prognosis for metastatic RCC is generally unfavorable. However, it has been observed in the literature that an accurate diagnosis of metastatic disease in its early stages can significantly enhance the survival rate. According to reported findings, the surgical removal of a solitary metastatic lesion of RCC after nephrectomy leads to a 41% survival rate at two years and a 13% survival rate at five years [8]. Unfortunately, the prognosis in patients with numerous metastases is much worse, with a five-year survival rate ranging from 0% to 7% [9].

## Conclusions

In conclusion, it is important to consider the possibility of metastatic RCC in individuals presenting with nasal or paranasal masses, particularly when accompanied by symptoms indicative of systemic involvement, such as hematuria. Timely detection of metastatic disease at an early stage can significantly mitigate perioperative complications and enhance overall survival rates.
